# Neural activity patterns stabilize during wakefulness and conscious experience

**DOI:** 10.1371/journal.pbio.3003252

**Published:** 2025-07-11

**Authors:** Simon van Gaal

**Affiliations:** Department of Psychology, Program Group Brain and Cognition, University of Amsterdam, Amsterdam, The Netherlands

## Abstract

Which neuronal markers can differentiate global brain states that underly states of consciousness? This Primer explores the findings of a new study published in PLOS Biology, which compares neural markers of loss of consciousness in flies when awake, asleep and anesthetized.

## Teaser

Several neuronal markers have been proposed to differentiate the global brain states that underly states of consciousness. A new pre-registered study in PLOS Biology compares neural markers of loss of consciousness in flies when awake, asleep, and anesthetized.

What causes us to lose consciousness during sleep or anesthesia? ‘Consciousness’, in its everyday use, refers to at least two related phenomena. Wakefulness or arousal (used interchangeably here), denotes variations in the organisms’ overall global state [1], for example being awake versus being asleep, in a coma, or anesthetized. Conscious experience (or conscious content) on the other hand, refers to all internal and external events that one is aware of, i.e., the phenomenal character of our experience. A sufficient arousal state is a prerequisite for conscious experience. For example, patients in coma cannot be awakened due to severe disruption to the brain’s arousal-regulating systems and (by definition) do not have conscious experience. When a sufficient arousal level is achieved, i.e., the system is awakened to a certain degree, arousal and experience often go hand in hand: the higher the arousal state, the higher the likelihood or richness of conscious experience [1]. Interestingly however, arousal and experience can also be dissociated, most strikingly observed in patients in the unresponsive wakefulness syndrome, previously known as the vegetative state [1,2]. In this condition, caused by severe brain damage, patients can be awake (they have their eyes open), but show no behavioral signs of conscious experience, for instance, revealed by the absence of coherent responses to external stimulation or the doctor’s command.

The neural mechanisms underlying transitions between different global states are a topic of active investigation in several species. In this domain, researchers often compare a high arousal state with a low arousal state (e.g., wakefulness versus sleep). Alternatively, arousal states may be varied gradually, for example by using different doses of a general anesthetic. This approach has resulted in several neural markers that can distinguish different global states, although many open questions remain [2,3]. A new study by Leung and colleagues [4] now goes beyond the current state-of-the-art in this field in several ways. First of all, the authors performed a preregistered report, in which they detailed all measurement protocols and analysis plans beforehand, followed by rigorous peer review before (confirmatory) analyses were performed. Second, the authors used a protocol in which they compare awake flies to not one, but two other global arousal states, namely sleep and anesthesia. This way, the authors could investigate the neural markers that commonly reflect the loss of consciousness in these different conditions. Third, the authors systematically investigated a large range of potential neural markers (>7,700), which minimized a selection bias of potential predictive markers for the loss of consciousness. Finally, the authors used rigorous cross-validation methods within and across multiple datasets, including a discovery (*N* = 13) and an evaluation set (*N* = 49) of flies, ensuring the reliability of the findings. The final results showed that the most predictive neural markers for a common loss of consciousness in the fly were related to the autocorrelation of neural time series, which reflect the brain’s capacity for stable and consistent information processing over time.

Although the data was obtained in flies during the recording of spontaneous neural activity (task-free condition), these findings resonate well with experimental findings from both task-free and task-based recordings in humans and other animals [2,3,5–7]. To illustrate, King and colleagues found that conscious visual perception in humans was also associated with a late, stable, and sustained neural signal, derived from across-time decoding analyses of magnetoencephalography data in human participants [5]. This stable activity pattern followed an initial more transient neural response, which reflected specific stimulus properties such as contrast, and was not related to stimulus visibility. That this late stable activity pattern is directly linked to conscious experience is highlighted by the fact that these stable representations are even observed when conscious content is hallucinatory, when our perceptual experience dissociates from the sensory input that was actually presented (e.g., seeing a house while a face stimulus was presented) [7]. A stable and long-lasting activity pattern is thus a common neural marker for high global arousal states (wakefulness) as well as conscious experience, across different species and conditions [3,4]. Therefore, it likely reflects neural commonalities at a “deeper level”. Indeed, these stable neural patterns are theorized to reflect the capacity of a neural system for (in the case of arousal state) or the occurrence of (in the case of experience) recurrent loops between distant brain regions [3,5–7]. Such recurrent loops allow neural activity to persist beyond initial stimulus presentation, which gives the brain time to integrate, amplify, and maintain information.

An interesting avenue for future research is to better link changes in the organism’s overall arousal state to conscious experience, not explicitly tested by Leung and colleagues. In the case of flies, this question is particularly challenging because it is debated whether flies posit (the possibility for) conscious experience in the first place. Further, testing for conscious experience in animals with simpler neural circuits than humans is generally difficult, though not impossible [8]. A possible strategy to link arousal state and conscious experience is to focus on shorter time scales. Critically, the arousal state of the brain may not only fluctuate slowly and globally, in the order of hours or days (e.g., sleep/anesthesia vs. wakefulness), but also more rapidly, within seconds or minutes, even during regular waking states [9]. A way to capture these faster fluctuations is by recording the size of the eye’s pupil in organisms that have pupils (e.g., flies do not). Pupil size has been shown to be a reliable physiological marker for (subtle) fluctuations in arousal, as it correlates with activity in arousal-regulating neuromodulatory sources [9]. Capitalizing on the pupil-arousal link is promising, because of the pupil’s high temporal resolution and the opportunity to acquire large numbers of trials per participant. Recent studies employing these methods in mice and humans have observed that optimal perceptual performance occurs at intermediate (pupil-linked) arousal states [9,10]. This suggests that arousal can indeed be too low, as also observed in patients in coma, but importantly also too high. Future work is still required to fully understand the complex interplay between arousal-mediated spontaneous activity and stimulus-evoked brain activity in shaping conscious experience, especially in these high aroused states.
